# Postoperative Rehabilitation to Improve Outcomes After Cervical Spine Fusion for Degenerative Cervical Spondylosis: A Systematic Review

**DOI:** 10.7759/cureus.39081

**Published:** 2023-05-16

**Authors:** Jeremiah Ling, Jeyvikram Thirumavalavan, Caleb Shin, Tiffany M Lee, Rex A. W Marco, Takashi Hirase

**Affiliations:** 1 Orthopedics and Sports Medicine, Houston Methodist Hospital, Houston, USA

**Keywords:** physiotherapy, physical therapy, rehabilitation, cervical spine fusion, degenerative cervical spondylosis

## Abstract

Postoperative rehabilitation has recently been identified as a high-priority research topic for improving surgical outcomes for degenerative cervical spondylosis (DCS). However, there remains no consensus on specific rehabilitation strategies. Thus, the objective of this study was to evaluate the effectiveness of postoperative rehabilitation strategies for short-term and long-term outcomes after cervical spine fusion for DCS. A systematic review was performed according to Preferred Reporting Items for Systematic Reviews and Meta-analysis (PRISMA) guidelines using the PubMed, Scopus, and Ovid Medline databases. All level I-IV therapeutic studies in the English language investigating the outcomes of postoperative rehabilitation strategies after cervical spine fusion for DCS were included. Nine studies with 895 patients with DCS (747 anterior-only fusion, 55 patients with posterior-only fusion, 93 patients with physiotherapy alone) were included in this analysis, with 446 (49.8%) patients receiving physiotherapy alone or standard postoperative therapy and 449 (50.2%) patients receiving standard postoperative therapy with additional intervention or augmentation. These interventions included pulsed electromagnetic field (PEMF) stimulation, telephone-supported home exercise program (HEP), early cervical spine stabilizer training, structured postoperative therapy, and a postoperative cervical collar. One level II study demonstrated that PEMF led to increased fusion rates at six months postoperatively compared to standard therapy alone, one level II study demonstrated that postoperative cervical therapy in addition to standard therapy was better than standard therapy alone in the improvement of neck pain intensity, one level IV study demonstrated home exercise therapy led to an improvement in neck pain, arm pain, and disability, and six level II studies reported no difference in clinical outcome measures between augmented or targeted therapy and standard postoperative therapy for DCS. In conclusion, there is moderate evidence to suggest that there is no significant difference in clinical and surgical outcomes between standard postoperative therapy and augmented or targeted postoperative therapy for cervical fusion in the setting of cervical spondylosis. However, there is some evidence to support that certain therapeutic modalities, such as PEMF stimulation, may lead to improved fusion rates, clinical outcomes, and patient satisfaction when compared to standard postoperative therapy protocols. There is no evidence to support a difference in effectiveness with different types of postoperative rehabilitation strategies between anterior and posterior fusions for DCS.

## Introduction and background

Degenerative cervical spondylosis (DCS) is an umbrella terminology comprising a variety of age and activity-related chronic degenerative pathologies of the cervical spine that may lead to progressive symptoms, including axial neck pain, radiculopathy, and spinal deformity [1]. Depending on how symptoms progress, conservative treatment or surgery is recommended [2]. Although a majority of the patients presenting with DCS can be treated non-operatively, with an improvement in symptoms, patients presenting with progressive deformities, neurologic deficits, instability, and debilitating symptoms that are unresponsive to non-operative management are common indications for operative management with cervical spine fusion [1-4].

Depending on the pathology’s specific clinical and radiologic characteristics, various techniques are used for the surgical treatment of DCS. Commonly utilized techniques include anterior cervical discectomy and fusion (ACDF), anterior cervical corpectomy and fusion (ACCF), cervical disc arthroplasty, posterior cervical decompression and fusion, and a combination of these anterior and posterior-based procedures [3,4]. Although recovery and gain of function are expected after surgical intervention, there is evidence that recovery can be incomplete and that patients can retain disabilities [5,6]. Physiotherapy or other targeted postoperative interventions may prove useful in optimizing postoperative recovery [2]. Due to the heterogeneity of clinical presentations and the specific techniques utilized to treat DCS, postoperative rehabilitation strategies are currently determined on a case-by-case basis and vary significantly depending on surgeon preference and the type of surgery performed.

Although postoperative rehabilitation has recently been identified as a high-priority research topic for improving surgical outcomes for DCS, there remains no consensus on specific rehabilitation strategies [7]. Standard postoperative therapy includes neck-specific exercises and general exercises, including ambulating, stair climbing, and non-impact cardiac exercises [2,4-6]. Possible augmented or targeted interventions includes pulsed electromagnetic field (PEMF) stimulation, telephone-supported home exercise program (HEP), early cervical spine stabilizer training, structured postoperative therapy, and a postoperative cervical collar [2,4-6].

Providers and patients face challenges and frustration when managing chronic neck pain like DCS [1]. Therefore, there is much importance in assessing if postoperative therapies can improve patient radiologic and clinical outcomes. Since this degenerative pathology is associated with increasing age, the prevalence of DCS is expected to rise [8]. Because postoperative rehabilitation interventions are so heterogeneous, evaluating if these interventions optimize care is essential. If so, targeted postoperative care should become a standard guideline for the treatment of DCS. Thus, the objective of this study was to perform a systematic review to evaluate the effectiveness of postoperative rehabilitation strategies for short- and long-term outcomes after cervical spine fusion for DCS.

## Review

Methods

Design and Search Strategy

The investigation was carried out and documented in accordance with the Preferred Reporting Items for Systematic Reviews and Meta-analyses (PRISMA) guidelines [9]. Two authors conducted independent searches on June 17, 2022, using the following medical databases: PubMed (1966-present), Scopus (1966-present), and Ovid Medline (1946-present). To ensure the search criteria were rigorous and encompassed applicable literature, a search protocol was developed by merging keywords such as "cervical," "fusion," "rehabilitation," and "therapy" using Boolean operators. Furthermore, a manual examination of the cited references was also conducted to decrease the inadvertent elimination of pertinent studies.

Eligibility Criteria

The analysis included all therapeutic studies published in the English language that investigated the clinical outcomes of postoperative rehabilitation strategies following cervical spine fusion for DCS and provided level I, II, III, and IV evidence according to the Oxford Centre for Evidence-Based Medicine (CEBM) criteria [10].

To avoid bias, the following types of studies were excluded from the analysis: non-cervical degenerative spondylosis studies, non-cervical spine fusion studies, cadaveric studies, basic science studies, animal studies, diagnostic studies, economic studies, prognostic studies, letters to editors, review articles, editorials, and surveys.

In cases where multiple studies from the same author(s) and/or institution(s) reported on the same or overlapping subjects, only the most recent study with the longest follow-up was included. However, data from the older studies were analyzed if not reported in the most recent study.

Quality Assessment and Data Extraction

Using a previously recommended methodology [11], two authors independently scrutinized all studies. In cases where two or more studies used the same patient population, the study with a longer follow-up, higher level of evidence, greater number of patients, and/or clearer methods and results was included. The Modified Coleman Methodology Score (MCMS) was used to assess the level of evidence (CEBM), study design, and methodological quality of each study [10-12]. Furthermore, the overall Strength-of-Recommendation Taxonomy (SORT) score and the Grading of Recommendations Assessment, Development, and Evaluation (GRADE) score were computed for all included studies [13,14]. Each study was evaluated for patient demographics (age, gender, diagnosis), re-operation rates, complication rates (major and minor), patient-reported outcome scores, radiologic outcomes, and overall conclusions, with data extracted from digital plots using the WebPlotDigitizer version 4.4 (Ankit Rohatgi, Pacifica, CA, https://automeris.io/WebPlotDigitizer) to best estimate the reported data, based on previously described methods [15,16]. Meta-analysis was conducted on outcome data without significant heterogeneity while a systematic review with a best-evidence synthesis was used as the synthetic review type for outcome measures with significant heterogeneity [17].

Statistical Analysis

The Statistical Package for the Social Sciences (SPSS) statistical software (Version 25.0; IBM Corp., Redmond, WA) was used to analyze the data. For continuous parameters, the mean and standard deviation (SD) were combined to calculate the weighted mean difference (WMD). Continuous data were analyzed using the two-tailed student's t-test while categorical data were analyzed using the chi-square test. Meta-analysis was conducted using the Revman 5.2 software (Cochrane Collaboration, Copenhagen, Denmark). A p-value ≤ 0.05 was regarded as statistically significant.

Risk of Bias Assessment

Each included study underwent a risk of bias assessment by two authors using the Revised Cochrane Risk-of-Bias Tool for Randomized Trials (RoB 2) and the Risk of Bias in Non-Randomized Studies of Interventions (ROBINS-I) assessment tool [18,19].

Results

Study Characteristics

**Figure 1:**
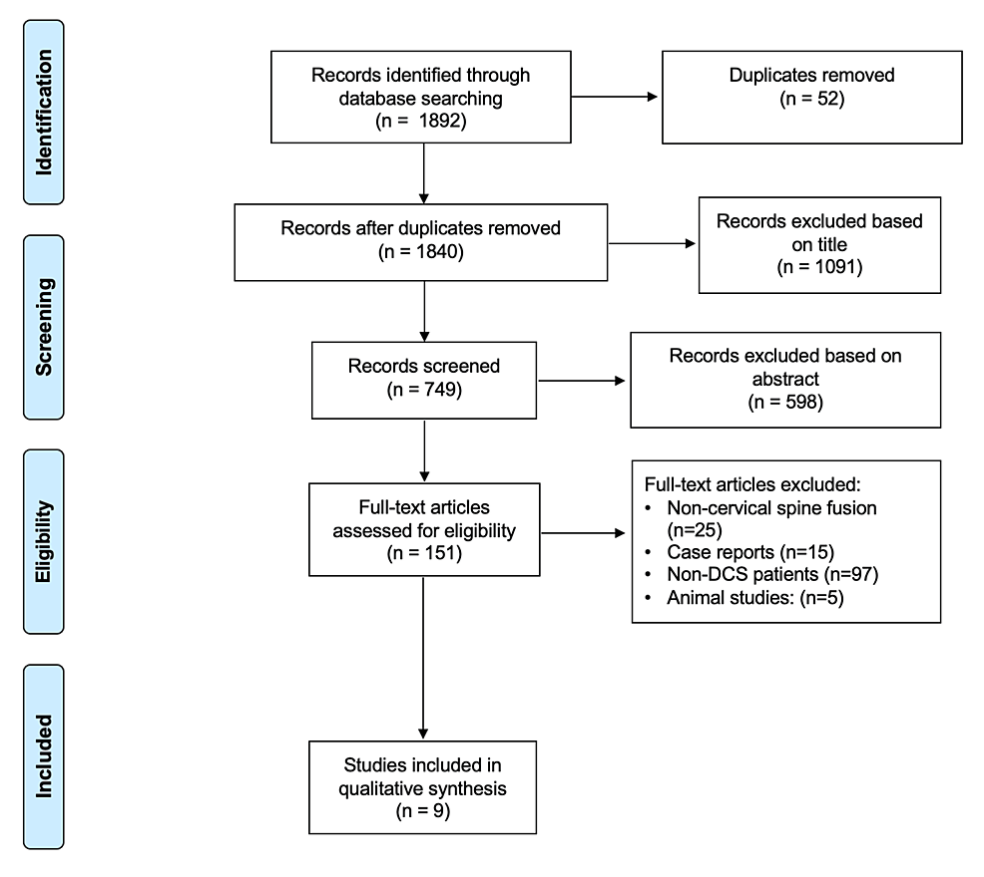
PRISMA Flow Diagram DCS = degenerative cervical spondylosis; PRISMA = Preferred Reporting Items for Systematic Reviews and Meta-Analysis

There were 1840 studies identified in our preliminary search, with nine studies that met inclusion criteria and were included in the study (Figure 1) [20-28].

Eight studies were level II evidence, and one was level IV. These nine studies contained 895 patients undergoing cervical spine fusion or non-operative management for DCS. There were 747 anterior-only fusions (83.5%), 55 posterior-only fusions (6.1%), and 93 with physiotherapy alone without surgery (10.4%). Out of the 802 patients that underwent cervical spine fusion for DCS, 353 (44.0%) received standard postoperative physical therapy and 449 (56.0%) received standard postoperative therapy and/or additional intervention or augmentation. These interventions included pulsed electromagnetic field (PEMF) stimulation, early cervical stabilization, postoperative home exercise programs, structured postoperative therapy, and a postoperative hard cervical collar (Table 1). According to MCMS, seven studies were rated as ‘good’ (scores between 70 and 84), and two were rated as ‘fair’ (scores between 55 and 69) [12]. The overall SORT (Surgical Outcome Risk Tool) score was A and GRADE (Grading of Recommendations, Assessment, Development, and Evaluation) score was B [13,14]. According to RoB2 and ROBINS-I, the overall risk of bias was low for seven studies and moderate for two studies (Table 1) [18,19].

**Table 1:** Study characteristics Rob2 = Revised Cochrane Risk-of Bias Tool for Randomized Trials; ROBINS-I = Risk of Bias in Non-Randomized Studies of Interventions; MCMS = Modified Coleman Methodology Score; ACDF = anterior cervical discectomy and fusion; PEMF = pulsed electromagnetic field; NR = not recorded

Study	Study design	Level of Evidence	Rob2 of ROBINS-I risk of bias assessment	MCMS	Treatment group(s)	No. of patients	Age mean ± SD (Range)	Male, n (%)	Smoker, n (%)
Coronado et al. [27]	Prospective Case Series	IV	Moderate risk	58 (Fair)	ACDF with Postoperative Physiotherapy	8	53 ± 15	3 (37.5%)	2 (25)
Engquist et al. [23]	Prospective Randomized Control Trial	II	Low risk	76 (Good)	ACDF with Postoperative Physiotherapy	31	49 ± 8	14 (45%)	8 (32)
					Physiotherapy Alone	32	44 ± 9	19 (59%)	9 (25)
Engquist et al. [24]	Prospective Randomized Control Trial	II	Low risk	76 (Good)	ACDF with Postoperative Physiotherapy	30	48 ± 8	13 (43%)	9 (33)
					Physiotherapy Alone	29	44 ± 9	17 (57%)	5 (17)
Foley et al. [20]	Prospective Multicenter Randomized Control Trial	II	Low risk	84 (Good)	No PEMF Stimulation	163	46.7 (26–72)	85 (53.1%)	79 (49.4%)
					PEMF Stimulation	160	46.9 (24–73)	90 (55.2%)	80 (49.1%)
McFarland et al. [26]	Prospective Randomized Control Trial	II	Low risk	72 (Good)	Usual Care Group	19	56.0 ± 9.8	10 (52.6%)	NR
					Early Cervical Spine Stabilizer Training Group	20	54.7 ± 10.5	6 (30%)	NR
Peolsson et al. [22]	Prospective Randomized Control Trial	II	Low risk	74 (Good)	Physiotherapy alone	32	46 ± 8.9	34 (54%)	17 (27)
					ACDF + physiotherapy	31			
Svensson et al. [28]	Prospective Multicenter Randomized Control Trial	II	Low risk	78 (Good)	Structured Postoperative Physiotherapy	51	49 ± 8	22 (43%)	NR
					Standard Postoperative Approach	55	49 ± 8	26 (47%)	NR
Abbott et al. [21]	Prospective Randomized Control Trial	II	Moderate risk	66 (Fair)	With Post-operative Cervical Collar Cage	17	53 ± 9	9 (53%)	NR
					Without Post-operative Cervical Collar Cage	16	69 ± 11	11 (69%)	NR
Wibault et al. [25]	Prospective Randomized Control Trial	II	Low risk	80 (Good)	Structured postoperative physiotherapy	101	50 ± 8	9 (53%)	49 (24)
					Standard postoperative approach	100		11 (69%)	

Radiologic Outcomes

Of the nine studies that met inclusion criteria, four studies with 423 patients reported radiologic outcomes (Table 2). Out of 323 patients who underwent ACDF for DCS in the prospective randomized study by Foley et al., 163 patients received standard postoperative care (soft cervical collar for one week) with PEMF, and 160 received standard postoperative care alone [20]. The PEMF group had a significantly higher fusion rate of 83.6% compared to the control group with 68.6% (p < 0.05) at six months postoperatively. Another study assessed a standard postoperative physical therapy program with or without the use of a rigid cervical collar. Out of 33 patients who underwent ACDF for DCS in the prospective randomized study by Abbott et al., 17 patients received a postoperative rigid cervical collar in addition to the standard postoperative physical therapy program for three weeks, and 16 received a standard postoperative physical therapy program alone for three weeks [21]. There were 100% fusion rates in both groups and no difference in sagittal malalignment at the three-month follow-up. In the prospective randomized study by Engquist et al., 59 patients with DCS and cervical monoradiculopathy were treated with either a physical therapy program alone (29 patients) or ACDF followed by postoperative physical therapy (30 patients) [24]. All 30 patients who underwent ACDF achieved adequate fusion at three months postoperatively. In a case series by Coronado et al., eight patients with DCS who underwent ACDF received postoperative physical therapy with an early telephone-supported home exercise program (HEP) and reported a 100% union rate at six months postoperatively [27].

**Table 2:** Radiologic outcomes ACDF = anterior cervical discectomy and fusion; PEMF = pulsed electromagnetic field; PT = physical therapy; HEP = home exercise program; NR = not recorded

	Foley et al. [20]		Abbott et al. [21]		Engquist et al. [24]		Coronado et al. [27]
Treatment group	ACDF + PEMF stimulation	ACDF only	ACDF + postop PT + hard cervical collar	ACDF + post-op PT	ACDF + post-op PT	PT alone	ACDF + post-op PT + HEP
No. of patients	163	160	17	16	30	29	30
Follow-up	6 months		3 months		3 months		6 months
Pts assessed for fusion at final follow-up, n (%)	122 (74.8)	118 (73.8)	17 (100.0)	16 (100.0)	30	N/A	30
Solid fusion, n (%)	102 (83.6)	81 (68.6)	12 (100.0)	12 (100.0)	30 (100.0)	N/A	30 (100.0)
Pseudarthrosis, n (%)	20 (16.4)	37 (31.4)	0 (0.0)	0 (0.0)	0 (0.0)	N/A	0 (0.0)
Other qualitative radiologic outcomes	PEMF led to significantly improved fusion rates at six months regardless of gender, age, smoking status, or number of operated levels		No difference in postoperative sagittal malalignment between the two treatment groups		NR		NR

Clinical Outcomes

All nine studies reported clinical outcomes (Table 3). In the study by Foley et al. that randomized patients to receive standard postoperative care (soft cervical collar for one week) with PEMF or standard postoperative care alone, there were no significant reported differences in visual analog scale (VAS) pain scores, 12-Item short form survey (SF-12), neck disability index (NDI), or adverse events between either group at the 12-month follow-up [20]. In the study by Abbott et al., patients received either a postoperative rigid cervical collar in addition to a standard postoperative physical therapy program for six weeks or a standard postoperative physical therapy program alone for six weeks [21]. Although both groups improved significantly in all outcome measures from baseline, the cervical collar group was associated with a statistically more significant reduction in neck pain and NDI compared to the group without a cervical collar with analysis of covariance. In the study by Peolsson et al., patients were assigned to a structured physiotherapy program or received anterior cervical decompression and fusion followed by the same therapy [22]. In both groups, there was significantly increased neck muscle endurance (p ≤ 0.01), manual dexterity (p ≤ 0.03), and right handgrip strength (p = 0.01). In the study by Engquist et al. (2013) patients with cervical radiculopathy were randomized to surgery with postoperative physiotherapy or physiotherapy alone [23]. The postoperative physiotherapy group reported significant improvement in their neck pain intensity (p = 0.039), a significant improvement in their global assessment of symptoms (p < 0.05), and 87% of postoperative patients described their symptoms as “better” compared to 62% in the physiotherapy alone group. Both groups reported a significant reduction in NDI, neck pain, and arm pain from baseline (p < 0.001). In another study by Engquist et al. (2017), patients received either postoperative physiotherapy following ACDF or physiotherapy alone without surgical intervention [24]. The group receiving ACDF followed by postoperative therapy had a greater reduction in NDI (p = 0.03) and a greater reduction in VAS score for neck pain (p = 0.01) compared to the group receiving physiotherapy alone [20]. In another study by Wibault et al., patients received either structured postoperative physiotherapy consisting of neck-specific exercises, graded resistance exercises, and isometric exercises or a standard postoperative approach [25]. The group receiving structured postoperative therapy had greater expectation fulfillment (p = 0.01) and greater enablement (p = 0.04) and reported less neck pain frequency (p = 0.05) than the group receiving only standard postoperative care. Both groups reported improvements in NDI and neck and arm pain intensity (p < 0.001). In the study by McFarland et al., patients post-ACDF were assigned to usual postoperative care or to an early cervical spine stabilizer training group [27]. Both groups reported significantly improved scores in the numeric pain rating scale (NPRS), and NDI from baseline. In the study by Coronado et al., eight patients received postoperative physiotherapy with an early telephone-supported HEP following ACDF [23]. Of these patients, six had reported significant improvement in catastrophizing their pain. Seven patients had significant improvement in NDI and NPRS scores for their neck and arm pain, greater than the minimal clinically important difference (MCID) of 2.6. Six patients reported a significant improvement in pain self-efficacy, greater than the MCID of nine points. For five patients, there was a significant improvement in fear of movement measured with the Tampa Scale of Kinesiophobia, greater than the MCID of four points. Three patients reported increased physical activity at the final follow-up. In the study by Svensson et al., patients post ACDF or PCF were assigned to a standard postoperative therapy or structured postoperative therapy group [28]. There was a significant improvement in headache intensity for both groups (p < 0.001).

**Table 3:** Functional outcomes ACDF = anterior cervical discectomy and fusion; PEMF = pulsed electromagnetic field; PT = physical therapy; HEP = home exercise program; f/u = follow-up; NDI = neck disability index; VAS = visual analog scale; NME = neck muscle endurance; NR = not recorded

Study	Surgery	Treatment group(s)	No. of patients	Final f/u, months (% available at final f/u)	NDI at 6 Weeks (SD)	NDI at 12 Weeks (SD)	NDI at 6 Months (SD)	NDI at 12 Months (SD)	VAS Neck Pain at 6 Weeks (SD)	VAS Neck Pain at 12 Weeks (SD)	VAS Neck Pain at 6 Months (SD)	VAS Arm Pain at 6 Weeks (SD)	VAS Arm Pain at 6 Months (SD)	VAS neck pain score reduction at final follow-up in mm (95% CI)	VAS arm pain score reduction at final follow-up in mm (95% CI)	NDI score reduction at final follow-up in % (95% CI)	NME flexion (ventral) improvement in seconds at 24 months	NME extension (dorsal) improvement in seconds at 24 months
Coronado et al. [27]	ACDF	Post-op HEP	8	8 (100)	30	NR	19	NR	7	NR	3	7	3	NR	NR	NR	NR	NR
Engquist et al. [23]	ACDF	Post-op PT	31	24 (100)	NR	NR	NR	NR	NR	NR	NR	NR	NR	32.0 (16.6-47.5)	18.1 (0.4-35.7)	14.2 (5.6-22.7)	NR	NR
	Non-op	PT Alone	32	24 (100)	NR	NR	NR	NR	NR	NR	NR	NR	NR	17.4 ( 2.2-32.6)	20.5 (3.2-37.9)	11.5 (3.0-19.9)	NR	NR
Engquist et al. [24]	ACDF	Post-op PT	30	>57 (100)	NR	NR	NR	NR	NR	NR	NR	NR	NR	39 (26-53)	33 (18-49)	21 (14-28)	NR	NR
	Non-op	PT Alone	29	>57 (100)	NR	NR	NR	NR	NR	NR	NR	NR	NR	19 (7-30)	19 (7-32)	11 (4-18)	NR	NR
Foley et al. [20]	ACDF	No PEMF Stimulation	163	12 (72.4)	NR	NR	23	22.8	NR	NR	NR	NR	NR	NR	3.6	NR	NR	NR
		PEMF Stimulation	160	12 (76.3)	NR	NR	31	25.6	NR	NR	NR	NR	NR	NR	3.4	NR	NR	NR
McFarland et al. [26]	ACDF	Standard Post-op Care	19	3 (100)	32.2 (14.2)	24.2 (12.0)	NR	NR	2.1 (1.5)	2.2 (1.6)	NR	NR	NR	NR	NR	NR	NR	NR
		Early Cervical Spine Stabilizer Training Group	20	3 (100)	24.6 (17.2)	16.9 (16.1)	NR	NR	2.0 (2.2)	1.7 (1.7)	NR	NR	NR	NR	NR	NR	NR	NR
Peolsson et al. [22]	Non-op	PT Alone	32	24 (100)	NR	NR	NR	NR	NR	NR	NR	NR	NR	NR	NR	NR	10	4
	ACDF	Post-op PT	31	24 (100)	NR	NR	NR	NR	NR	NR	NR	NR	NR	NR	NR	NR	14	30
Svensson et al. [28]	ACDF or PCF	Post-op PT	51	12 (64.7)	NR	NR	NR	NR	NR	NR	NR	NR	NR	NR	NR	NR	NR	NR
		Standard Post-op Care	55	12 (65.5)	NR	NR	NR	NR	NR	NR	NR	NR	NR	NR	NR	NR	NR	NR
Abbott et al. [21]	ACDF	Post-operative Hard Cervical Collar	17	24 (47.1)	27.99 (1.5)	NR	24.7 (0.2)	23.65 (0.4)	NR	NR	NR	NR	NR	3.19	2.29	7.94	NR	NR
		No Cervical Collar	16	24 (43.7)	32.91 (2.7)	NR	28.07 (1.9)	28.17 (0.8)	NR	NR	NR	NR	NR	2.73	1.73	9.93	NR	NR
Wibault et al. [25]	ACDF or PCF	Post-op PT	101	6 (88.1)	NR	30.6 (18.1)	24.5 (18.5)	NR	NR	NR	NR	NR	NR	34.6	36	17.5	NR	NR
		Standard Post-op Care	100	6 (81.0)	NR	27.0 (16.0)	22.3 (17.3)	NR	NR	NR	NR	NR	NR	33.9	29.3	21	NR	NR

Discussion

Cervical fusion is a common and effective surgical procedure used for cervical spondylosis, which includes axial neck pain, radiculopathy, myelopathy, and spinal deformity when non-operative management is inadequate [29-31]. After cervical fusion surgery for DCS, the most effective postoperative rehabilitation protocol remains uncertain. In addition, there is a lack of consistency in the type of postoperative therapy provided across different institutions. Despite multiple comparative clinical outcome studies in the literature, this systematic review is the first to compare specific, targeted postoperative rehabilitation protocols with the current standard of care. The hypothesis being tested is that there is no significant difference in postoperative outcomes between standard postoperative therapy and targeted therapy protocols for patients who have undergone cervical fusion surgery for cervical spondylosis. The study found moderate evidence to suggest that there is no significant difference in postoperative outcomes between standard postoperative therapies and augmented or targeted therapy for patients who have undergone cervical fusion surgery for cervical spondylosis. However, the study did find some evidence to support the use of pulsed electromagnetic field (PEMF) therapy as an adjunct to standard postoperative therapy, which may significantly improve outcomes. Moreover, there was moderate evidence to suggest that augmented therapy, which is individualized and targeted to the patient's needs, can improve a patient's overall assessment of their condition. Two studies showed that structured postoperative therapy improved the individual rating of a patient's condition after surgery. Additionally, structured therapy was found to be effective in reducing neck pain and increasing the patient's sense of enablement and fulfillment compared to standard therapy alone. Overall, these findings suggest that while there may not be a significant difference in outcomes between standard postoperative therapy and targeted therapy protocols, the latter can have positive effects on patient satisfaction and well-being. Further research is needed to determine the optimal rehabilitation protocol for patients following cervical fusion surgery.

The study found no significant difference in VAS pain scores, SF-12, NDI, or neck/arm pain intensity ratings between augmented or targeted therapy and standard postoperative therapy alone. Both interventions significantly improved symptoms from baseline in most of the studies, with no inter-group significant difference. Current evidence demonstrates that any therapy is superior to no intervention postoperatively for cervical spondylosis patients following cervical fusion [32]. This systematic review, demonstrating equivocal improvement in patient outcomes between augmented or targeted therapy and standard postoperative therapy for patients with cervical spondylosis following cervical fusion, is consistent with the current evidence [32].

Postoperative therapy has been demonstrated to improve outcomes without deleterious effects on instrumentation in cervical fusion [32]. In this systematic review, augmented and targeted therapies improved patient catastrophizing and satisfaction postoperatively, particularly in the Coping Strategies Questionnaire - Catastrophizing Scale (CSQ-CAT) score. PEMF was shown to increase fusion rates postoperatively for DCS compared to physical therapy alone [33]. For some targeted postoperative interventions like cervical spine collars, evidence has shown its use makes no difference in clinical outcomes [34]. However, Abbott et al. also utilized rigid cervical collars and although there was no significant difference in radiologic outcomes, the rigid collar had a statistically larger reduction in neck pain and NDI compared to the group without a cervical collar. In this review, while most of the studies support that postoperative therapy benefits patients following cervical fusion, as both standard postoperative therapy and augmented or targeted therapy led to significant improvement in VAS pain scores, SF-12, NDI, or neck/arm pain intensity ratings from baseline, the presence of conflicting results points to a gap that the success rate of many postoperative targeted therapies has yet to be truly elucidated.

There are several limitations to this review. First, despite the inclusion of nine comparative studies, there was a relatively limited number of patients analyzed. Furthermore, the heterogeneity of endpoints in each study, including study subjects (i.e., catastrophizing scale, neck and arm pain intensity, and range of motion metrics), use of controls, and methods used to evaluate clinical outcomes limited direct comparisons of results. Limited studies were directly comparing postoperative standard intervention versus postoperative with tailored interventions and no papers compared the tailored therapies to each other. Furthermore, there were no included articles that directly compared postoperative rehabilitation to no rehabilitation at all, which would have served as a useful negative control in this population. Finally, the stringent search protocol and limiters may have excluded other relevant studies on this topic, including those published in non-English or for pediatric populations.

Despite these limitations, to our knowledge, this is the most comprehensive systematic review to date examining the effectiveness of various postoperative rehabilitation strategies for both short- and long-term outcomes following cervical spine fusion for degenerative cervical spondylosis (DCS). Nevertheless, more research is necessary to determine the most effective rehabilitation modality for patients who have undergone this surgery. One important area for future research is the comparison of different postoperative rehabilitation strategies against a control group receiving standard postoperative surgical care. Such comparisons will help identify the most effective therapy for patients with severe DCS who have undergone surgery. It is important to note that this type of comparison is crucial for establishing an optimal standard of care for all patients suffering from DCS. Furthermore, the development of postoperative guidelines to determine the best intervention for each patient will help establish an improved standard of care. Such guidelines should be tailored to the specific needs and goals of individual patients, taking into account factors such as age, overall health, and extent of the surgical procedure. Finally, future work should focus on disseminating standard rehabilitation programs post-surgery. This will help ensure that patients receive the highest quality of care and have the best possible outcomes after surgery. By implementing such programs, healthcare providers can improve patient outcomes, reduce healthcare costs, and improve the overall quality of life for those suffering from DCS who have undergone cervical spine fusion surgery.

## Conclusions

There is moderate evidence to suggest that there is no significant difference in clinical and surgical outcomes between standard postoperative therapy and augmented or targeted postoperative therapy for cervical fusion in the setting of cervical spondylosis. However, there is some evidence to support that certain therapeutic modalities, such as PEMF stimulation, may lead to improved fusion rates, clinical outcomes, and patient satisfaction when compared to standard postoperative therapy protocols. There is no evidence to support a difference in effectiveness with different types of postoperative rehabilitation strategies between anterior and posterior fusions for DCS.
